# Development and validation of a new method for visual acuity assesment on tablet in pediatric population: eMOVA test

**DOI:** 10.1186/s12886-022-02360-8

**Published:** 2022-04-19

**Authors:** Noémie Stoll, Elsa Di Foggia, Claude Speeg-Schatz, Hélène Meunier, Adam Rimele, Pascal Ancé, Pierre-Henri Moreau, Arnaud Sauer

**Affiliations:** 1 Three borders ophthalmologic center, 76 rue de Battenheim, 68170 Rixheim, France; 2 Colmar Civil Hospitals, 39 avenue de la Liberté, 68000 Colmar, France; 3 University professor, University hospitals of Strasbourg, 1 quai Louis Pasteur, 67000 Strasbourg, France; 4 Cognitive and Adaptative Neuroscience Laboratory, Strasbourg University, 67000 Strasbourg, Alsace France; 5 SILABE Platform, Strasbourg University, Fort Foch, 67207 Niederhausbergen, France

**Keywords:** Amblyopia, Visual screening, Visual acuity, Pediatrics ophthalmology, Child, eMOVA test, Tablet

## Abstract

**Background:**

Amblyopia is a major public health concern. Its screening and management require reliable methods of visual acuity assessment. New technologies offer nowadays many tests available on different app stores for smartphone or tablet but most of them often lack of scientific validation for a medical use. The aim of our study was to attempt validating a tablet-based near visual acuity test adapted to the pediatric population: the eMOVA test (electronic Measurement Of Visual Acuity) by comparing visual acuity measured with more conventional test.

**Methods:**

A cohort of 100 children aged 3 to 8 attending the ophthalmic-pediatric for eye examination between September 2016 and June 2017 were included in the study. Near visual acuity was assessed on participants using both the eMOVA test and a Standard test (Rossano-Weiss test). Duration of each test, its comprehension, its acceptability and the attention of the child during the test was also investigated.

**Results:**

The eMOVA test overestimated near visual acuity by 0.06 logMAR. This difference, statistically significant, was not clinically relevant. The duration of the eMOVA test was longer than the reference test, but less discomfort and preferred by children and their parents compared to standard tests.

**Conclusion:**

The eMOVA test appears as a reliable test to assess near visual acuity in children. By its portability and efficiency, this application proved to be a relevant tool to be used for children eye examination in daily routine at the hospital.

## Background

Amblyopia also mentioned as the "Lazy Eye" is a vision disorder considered with refractive errors as the commonest causes of visual impairment among children. Arnold and collaborators reported in a recent study that one out of 40 preschool-aged children are affected and one out five children are at high risk for developing amblyopia. [1] Most causes of amblyopia are treatable, provided they have been detected early enough. [2] If amblyopia is detected during the childhood, loss of vision can be easily prevented. Indeed, during the development of the eye and visual pathway at a young age, it is observed a period considered as "sensitive" during which the maturation of the visual pathways is not yet fully completed [3]. As a result, a proper management and treatment of amblyopia before the end of this period can partially or completely reverse the progression of the disorder.

In general practice routine, when a child is diagnosed with amblyopia, the appropriate care follows the following steps. At first, the management will be essentially etiological with in some cases the need for a surgery. In a second step, the refraction error is assessed under cycloplegia in order to provide an optical correction adapted to the deficit. Finally, a reeducation to treat amblyopia can be started with an optical penalization [3].

Effective screening for these disorders is therefore necessary and should be sought as early as possible. This screening, which is a public health priority, requires assessment methods adapted to the child and the disorders sought, ranging from mass screening to ophthalmic-pediatric consultation.

The VIP study investigated the utility of traditional tests used in screening children 3 to 5 years old. No test was optimal but non-cycloplegic retinoscopy, self-refraction by Retinomax or Suresight Vision Screener and the Lea Symbols scale showed the best performance [4].

Nowadays standard assessment methods for measuring visual acuity are varied and none of them includes all the characteristics requested for an ideal test [5–7].

A visual acuity test would have to include all the critical parameters and methods needed for an accurate assessment. It should allow measuring angular acuity, presenting a logarithmic progression scale during the assessment. Moreover, the space between optotype should be equal to the size of the optotype presented and the space between lines should be equal to the size of the optotype. The test should present to the patient a sufficient number of optotype and include confusion’s letter at the beginning and at the end of the assessment. To finish the luminance should be comprised between 150-650 cd/m^2^ and the contrast superior to 70 % [5–7]. Thus, grouping all these characteristics make complex the possibility to obtain a complete test.

For more than a decade, the development of new technologies is constantly improving and would be a possibility to overcome these technical difficulties. Moreover, the demand for the use of new technologies in the medical field is increasing and pushing practitioners to evolve in the renewal of their equipment and assessment routine [8]. In 2011, WHO defined the term "mHealth" as a medical and public health practice supported by mobile devices such as mobile phones, patient monitors, PDAs, and other wireless devices   [8]. In the ophthalmology field, new digital tools have been developed to take in consideration diabetes [9] and AMD [10] for eye checks. Another example is the development of smartphone camera adapters permitting to get accurate pictures of the anterior and posterior eye segments [11] as well as the study of contrast sensitivity and color vision [12, 13]. Because of their portability, these new technologies turned to be particularly relevant for eye check improvement in developing countries [14]. In the field of pediatric ophtalmology, some authors have proposed a binocular approach to amblyopia with rehabilitation exercises in the form of games on iPad with encouraging results [15–19].

In this context, we developed an application for tablet through the corporation regrouping the Ophthalmology Department of Strasbourg University Hospital and Strasbourg University permitting to improve visual screening in the pediatric population. The aim of our study was to assess the reliability of a modern near visual acuity test compared to more standard methods on children's near visual acuity aiming to provide an effective tool for screening visual disorders.

## Materials and methods

### Ethical approval

The study and data collection were conducted in accordance with all local laws and were compliant with the principles of the Declaration of Helsinki. The study was approved by local institutional review board such as Strasbourg University and Strasbourg University Hospital Center Ethics Committees.

### Touchpad and Application characteristics

The tablet chosen was an Android Lenovo Tab2 A10-30 equipped with a 10.1 inches touchscreen offering sufficient features such as resolution quality and contrast to measure near visual acuity. The application eMOVA (electronic Measurment Of Visual Acuity) has been developed by engineers and researchers working at Strasbourg University to be used on an Android operating system to assess near vision.

**Fig 1:**
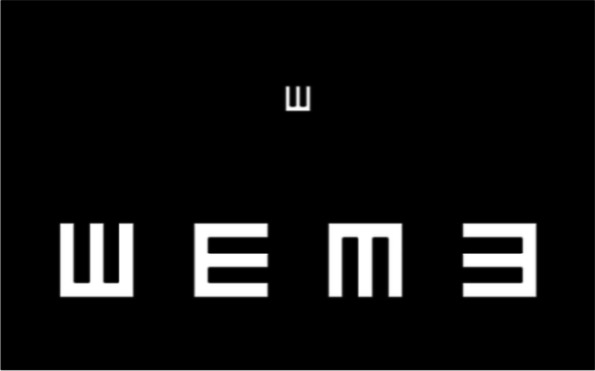
eMOVA test screen

The visual acuity test chosen to be used with the application was Raskin's E isolated optotypes (Fig. 1). This choice was motivated by the desire to be able to use the same optotype with the eMOVA test and the Rossano-Weiss test. Indeed, the conventional tests used at the Strasbourg University Hospital in current practice includes mainly the use of the Rossano-Weiss test to assess children near visual acuity. Drawings are usually used for preverbal children as well as numbers and Raskin’s E when it is possible. However, we considered to be preferable using a method permitting a visual acuity assessment based on the minimum resolution angle what permits the Raskin’s E. The size of each optotypes presented on the tablet had to be equivalent to what is presented with the Rossano-Weiss test. The size of the tablet optotypes being dependent on the size of a pixel, we then have calculated and developed a sequence close as possible to that purpose with an equivalent size match.

### Participants eligibility

The study was conducted with Children coming for eye examination at the Ophtalmology Pediatric Department of Strasbourg University. A total of 100 children were included in the study from September 2016 to June 2017.

#### Inclusion criteria

Common inclusion criteria were considered for this study such as (i) children had to be aged 3 – 8 years inclusive, (ii) child had to be able to express and prove his agreement to the tests and (iii) parents or legal representative had to consent the participation of their child to the test.

#### Exclusion criteria

Children were not recruited if they had already performed once the eMOVA test before the session. Moreover, children presenting with a disability suggesting that assessment of visual acuity was not possible were not included in the study.

### Study procedure

This monocentric study aimed to compare the near visual acuity scoring obtained with the eMOVA test and the Rossano-Weiss reference test. Both tests were conducted blindly by two different practitioners during the same eye examination and under the same environmental conditions. The tests were administrated by one ophthalmologist and three orthoptists trained for both tests. The first test to be presented to the child was randomly selected according to the order of children arrival for eye examination. After the completion of the first test, the child was remaining installed before the second practitioner started the second test. A sequence effect was sought to verify the absence of bias being potentially linked to the order of test presentation because of concentration and fatigability being considered as important factors in pediatric clinics. The right eye was the first eye tested for all children.

A distance of 40 cm between the eyes of the child and the screen of the tablet was kept. Since accommodation is dependent on reading distance, it is very important to adopt a fixed distance when assessing near visual acuity. The American Academy of Ophthalmology recommends a distance of 35 to 40 cm for assessing near visual acuity [20]. When a child watches a test at a distance self-chosen (between 5 and 20 cm), the visual acuity measured decreases by 0.15 logMAR compared to the visual acuity measured at 40 cm. At 40 cm distance, the influence of accommodation is minimal and permit easily to compare near and distance visual acuity measurements. Moreover, no evidence has shown a difference between near and distance visual acuity in children presenting a normal or reduced vision when the distance is kept at 40 cm. At 4 year old, children are fully able to maintain this distance when measuring near vision [20].

### Evaluation criteria

The principal study outcome was the measurement of near visual acuity presented in log and decimal notation. The secondary outcomes considered were: the understanding of the test, the child's attention when carrying out the test, the respect of the distance of realization of the test, the duration of the test, the child’s anxiety during the test.

The understanding of the test and the child's attention were subjectively evaluated from 0 to 5 by the examiner, corresponding to a very bad, bad, average, good, very good understanding or attention. The distance was checked using a measuring tape and the examiner gave a score from 1 to 5 according to the same methods as for the previous criteria. The duration of the test was measured using a stopwatch and the anxiety during the test was assessed using the FLACC scale ( Face Legs Activity Cry Consolability) [21–23]. The weekly time duration on tablet or smartphone by the child was also collected as well as the number of previous eye examination to find out if their habit of using this technology could influence the outcome. A reproducibility test of the eMOVA test was performed on a sample of thirty eyes, independent of the main study. For this, the test was carried out twice in a row under identical conditions.

### Statistical analysis

The analysis of the results was carried out in several stages. First, we calculated the average near visual acuity obtained with each of the two tests as well as the average difference in near visual acuity obtained between the two tests for each participant.

In order to test the equivalence of both near visual acuity tests, we carried out in a second step a concordance study for the main judgment criterion analysis. The concordance analysis for the quantitative variables were performed graphically using the Bland & Altman method and calculated with the intra-class correlation coefficient (ICC). For qualitative variables, the concordance was evaluated by calculating the Kappa coefficient. The 95% confidence intervals of these two indicators were calculated using a bootstrap resampling method. A Deming regression was performed for the measurement of near visual acuity in order to search if there was some proportional or systematic bias.

Third, a superiority study was conducted for the analysis of secondary endpoints to compare quantitative variables using Wilcoxon signed rank test and for qualitative variables the Bhapkar test.

A sequence effect was finally tested by comparing near visual acuity according to the sequence of realization with a signed rank test of Wilcoxon to identify if the performances obtained with one test were influenced by the previous realization of the other test.

A p-value <0.05 was considered statistically significant. The analysis were performed using R version 3.2.2 software.

## Results

### Patients characteristics

One hundred patients were included between September 2016 and June 2017. The average age was 68 months. Four children were excluded from the analysis for not being able to perform the tests. Characteristics of the 96 patients included are presented in Table 1. Amblyopia was defined as a monocular acuity less than or equal to 6/10 or with a difference in visual acuity greater than or equal to 2/10 between both eyes. Children with uni or bilateral amblyopia were included as amblyopic. Any absence of emmetropia was recorded as myopia, hyperopia or astigmatism.

**Table 1:** Patients characteristics

Criteria collected	Number (Total 96)
Age : (in months)	
-Between 36 and 59	28 % (27/96)
-Between 60 and 66	22 % (21/96)
-Between 67 and 79	23 % (22/96)
-Between 80 and 112	24 % (23/96)
Sex :	
-Girls	56 % (54/96)
-Boys	44 % (42/96)
Number of previous eye examination:	
-First examination	16 % (15/96)
-Between 1 and 5	38 % (36/96)
-Between 6 and 10	21 % (20/96)
-> 10	25 % (24/96)
Reason for examination	
-Screening	25 % (24/96)
-Amblyopia or known risk factor of amblyopia	75 % (72/96)
Diagnosis :	
Known amblyopia	12 % (12/96)
Strabismus	34 % (33/96)
Hypermétropia	22 % (21/96)
Astigmatism	10 % (10/96)
Myopia	4 % (4/96)
Time duration on smartphone or tablet at home per week	
-< 3 hours	62 % (60/96)
-between 3 and 7 hours	22 % (21/96)
-between 7 and 14 hours	13 % (12/96)
-> 14 hours	3 % (3/96)

### Principal outcome

The mean near visual acuity using the Rossano-Weiss test was -0.22 logMAR or 6.2/10 for the right eye and -0.24 logMAR or 6.1/10 for the left eye. When assessed with the eMOVA test, mean near visual acuity was -0.28 logMAR or 5.9/10 for the right eye and -0.24 logMAR or 6.1/10 for the left eye. The mean difference in near visual acuity measured between both tests was -0.06 logMAR or 0.3/10 for the right eye and -0.01 logMAR or 0/10 for the left eye. These data related to the principal study outcome are presented in Table 2. The maximum near visual acuity achieved with both tests was -0.18 logMAR. The minimum near visual acuity assessed was -0.67 logMAR and -1.2 logMAR, respectively, for the Rossano-Weiss test and the eMOVA test. The first quartile, median, third quartile and mode were -0.18 logMAR for all groups.

**Table 2:** Primary outcome main results presented in logMAR (collected from the 96 patients included)

	Right eye series	Left eye series
Mean visual acuity (in logMAR)		
-Rossano-Weiss test	-0.22	-0.24
-eMOVA test	-0.28	-0.24
Mean difference between both tests	-0.06 [-0.48 – 0.36]	-0.01 [-0.40 – 0.38]
*p*	0.006 [-0.10 – 0.02]	0.006 [-0.10 – 0.02]
Correlation coefficient	0.40	0.43

Overall, the differences in near visual acuity using both tests were very limited. In order to estimate the equivalence of both tests, a statistical analysis of concordance was conducted.

For this analysis, both sets of values ​​were analyzed separately to verify the validity of our results for each eye: first values measured for the right eye and secondly the one for the left eye of each participants. The correlation coefficient r for visual acuity measurement obtained with the Rossano-Weiss test and the eMOVA test was respectively 0.40 (*p* <0.001 and 95 [0.21-0.55]) for the right eye series and 0.43 (*p* <0.001 and IC 95 [0.26-0.58]) for the left eye series (Table 2).

When near visual acuity measured with the Rossano-Weiss test was -0.18 logMAR, 91% of visual acuity scores obtained for the right eyes and 95% of the scores obtained for the left eyes were comparable with the measured obtained with the eMOVA test.

The mean difference between both tests was -0.06 logMAR (lower bound: -0.48, upper bound 0.36) for the right eye series and -0.01 logMAR (lower bound: -0,40, upper bound: 0.38) for the left eye series.

**Fig 2:**
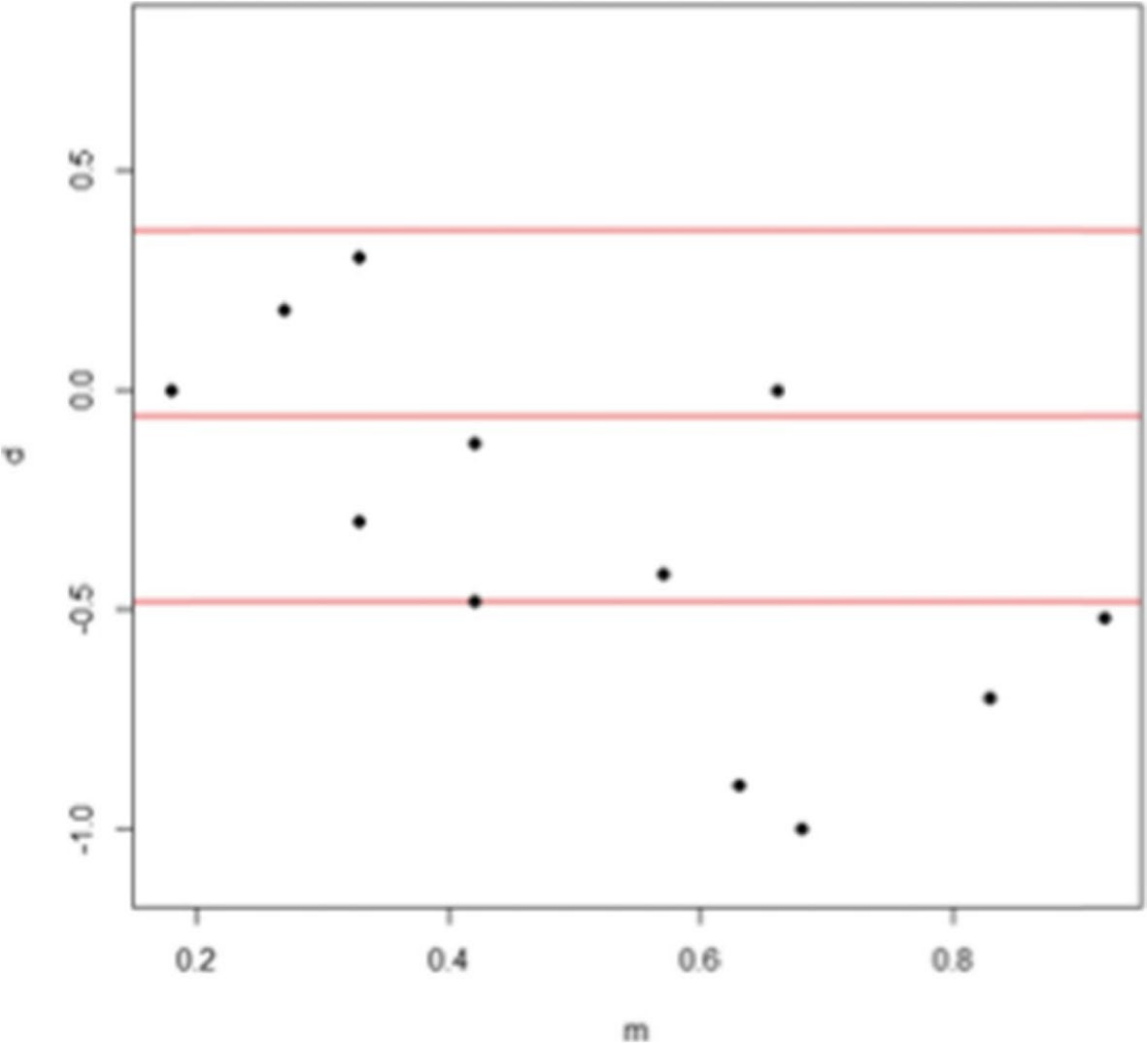
Bland and Altman analysis: quantitative concordance analysis for the visual acuity of the right eye (in logMAR)

On the Bland and Altman analysis (Fig. 2), concordance was very good for the highest near visual acuities. However, for near visual acuity scores lower than -0.6 logMAR, the concordance became less good. The eMOVA test showed better near visual acuity measurements compared to the Rossano-Weiss in participant with a low near visual acuity.

**Fig 3:**
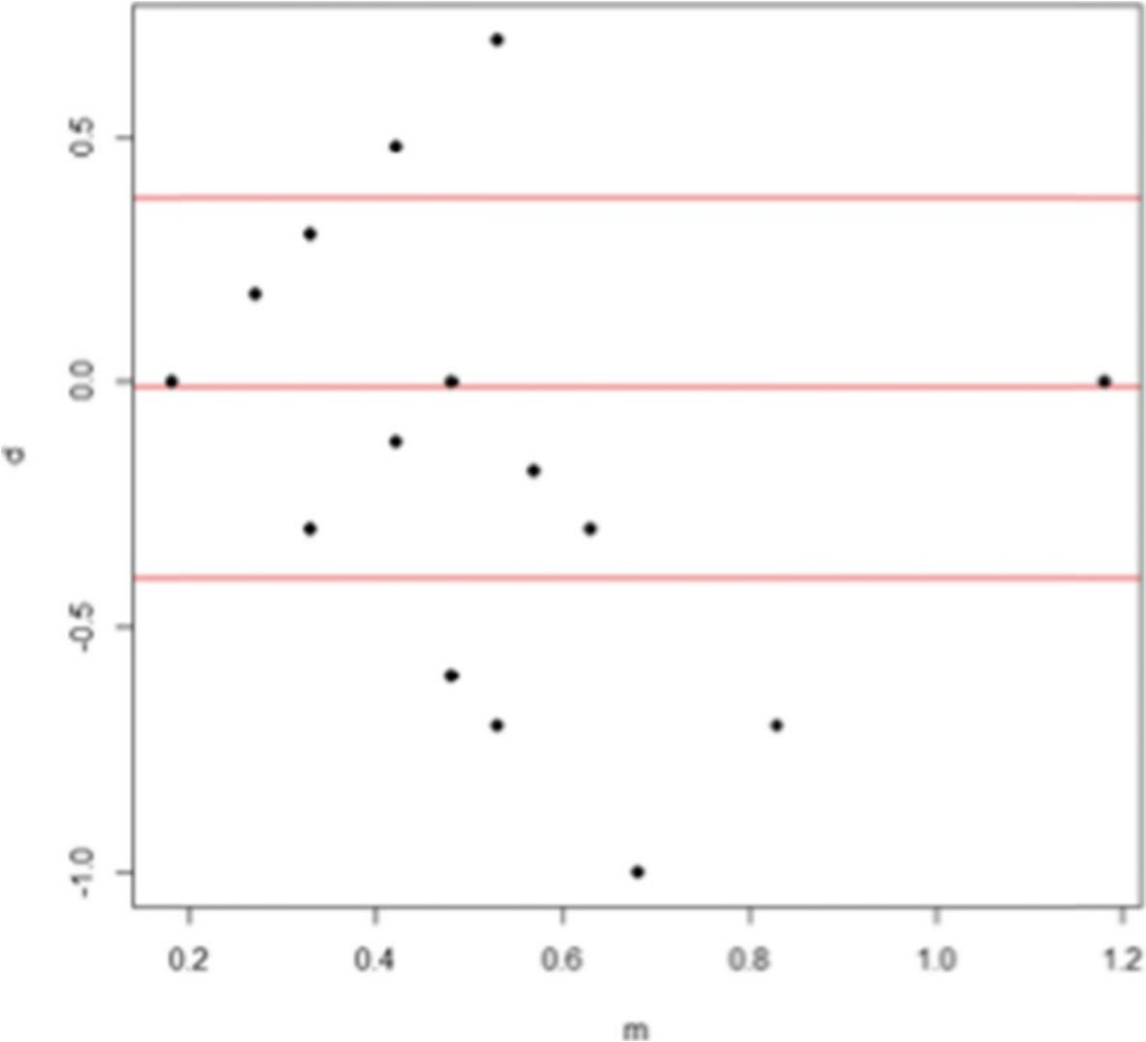
Bland and Altman analysis : quantitative concordance analysis for the visual acuity of the left eye (in logMAR)

The eMOVA test showed near visual acuities 0.06 logMAR higher than that measured with the Rossano-Weiss test for the right eye series and near visual acuity scores of 0.01 logMAR for the left eye series. When using the lower and upper limits, the difference in values ​​obtained between both tests was at most -0.48 logMAR for the right eye series (Fig. 2) and -0.40 logMAR for the left eye series (Fig. 3).

The mean difference between both tests was -0.06 logMAR in favor of the eMOVA test using the Wilcoxon test. This difference was statistically significant (*p* = 0.006). The 95% confidence interval of this difference was [-0.10 - 0.02]. These results were the same for both series. This means that at most, the difference in near visual acuity measured between the two tests would be 0.10 logMAR.

Both analysis, concordance and superiority tests, showed the same tendency: the eMOVA test overestimated near visual acuity statistically. However, this difference was not clinically relevant as it would be at most 0.06 logMAR.

Analysis of the Bland and Altman graphs showed that measurement concordance decreases when visual acuities are low. Indeed, the concordance was good for the visual acuities comprised of 0 and -0.6 logMAR and became lower for visual acuities lower than -0.6 logMAR (Fig. 2). This trend was also observed for the second series (Fig. 3).

The results of the Deming regression was: Intercept = 0.14 CI 95 [0.10; 0.18], Slope = 0.28 CI 95 [0.09; 0.47] for the series on the right eyes and Intercept = -0.11 CI 95 [-0.76; 0.54], Slope = 1.64 CI 95 [-1.91; 5.19] for the series on the left eyes.

These results show the absence of systematic and proportional bias for the series on the left eyes. Contrary to these results, systematic and proportional biases are found for the series on the right eyes. However, these biases have no clinical relevance because the average difference found between the near visual acuity measurements taken with the eMOVA test and with the Rossano-Weiss test is 0.01 on the Bland et Altmann, which has no clinical consequence.

### Secondary outcomes

Results related to secondary outcomes are presented in Table 3. Eighty-four percent (84%, 81/96) of all children had perfectly understood the testing conditions with a score of 5. Eighty-one percent (81%, 78/96) of all children presented with an attention score of 5. Eighty-four percent (84%, 81/96) of all children had a distance score greater than or equal to 4. No statistical difference has been observed when the comparison has been performed between both tests.

**Table 3:** Comparison of secondary outcomes scores (collected from the 96 patients included)

	Rossano-Weiss	eMOVA	p
Understanding	4.8	4.8	0.11
Attention	4.7	4.8	0.26
Respect of distance	4.4	4.3	0.72
Duration	43	64	<0.001
Total FLACC	0.3	0.1	0.01

The mean difference in assessment time duration between both tests was 21 seconds for both eyes checks. The minimum duration was 11 seconds for the Rossano-Weiss test and 23 seconds for the eMOVA test. The maximum duration was 340 seconds for the Rossano-Weiss test and 174 seconds for the eMOVA test. These differences were statistically significant (*p* <0.001).

Overall, we noticed that child's anxiety increased across the testing procedure for both tests. The average FLACC scores obtained before / during / after the test were all lower with the eMOVA test. The test was felt by the children less stressful when performed with the tablet than the Rossano-Weiss test and this difference was statistically significant (*p* = 0.01).

### Testing parameters

The reproducibility of the eMOVA test was calculated on 30 eyes check samples (both eyes of 15 children) which has been performed twice in a row under identical conditions. The reproducibility of the eMOVA test was very good with an intra class correlation coefficient of 0.93 (95% CI [0.87-0.97]). The Rossano-Weiss test was used in our study as the standard for comparison with eMova test. We have defined as amblyopic any child with a monocular acuity at the Rossano-Weiss test less than or equal to 6/10 or with a difference in visual acuity greater than or equal to 2/10 between both eyes. The sensitivity observed for the eMOVA test was 56% and its specificity 88%. The negative predictive value of the test was 90% and its positive predictive value 53%. The accuracy of the eMOVA test was 82%. This parameters was obtained with the values presented in Table 4.

**Table 4:** Distribution of true positives, true negatives, false positives and false negatives ( M+ corresponds to the presence of amblyopia and M- to its absence)

		**eMOVA**		
		M+	M-	Total
**Rossano-Weiss**	M+	10	8	18
	M-	9	69	78
	Total	19	77	96

#### Test preference

After performing the two tests, the second examinator asked the parents which one of the two tests they would prefer to use for a future examination. The eMOVA test was mostly chosen by parents and children. Eighty-seven percent (87% or 84/96) of the children and 80% (or 77/96) of the parents would choose the eMOVA test.

#### Sequence effect

In order to know if the child's concentration, the length of the examination or the performance of a previous test could affect our results, we looked for a sequential effect.

No statistical difference regarding the testing order between both tests has been observed after analysis which limits potential sequential bias in our study (*p*=0.61).

## Discussion

Our study showed the interest of the eMOVA test as a screening test for amblyopia in children from 3 to 8 years old. It showed its reliability with a correct agreement with the Rossano-Weiss test (ICC 0.43), 95% correspondence for the best near visual acuity and a mean difference in near visual acuity of 0.06 logMAR between the two tests. These results are good compared to those of studies that have developed similar tests [24, 25]. The eMOVA test is reproducible (ICC 0.93) and simple. Indeed the understanding of the test was high (score of 4.8/5) and the testability of 97% in our sample which is very good in comparison with the main tests having shown their good performance [26, 27]. However, this comparison should be made with caution in view of the variability of the ages present in the samples presented by this studies. Studies of only children under 10 are not the most common [28–35]. Their comparison must therefore be careful but tends to show a faster test duration with the eMova test which can favor the child's concentration and therefore the reliability of the measurement of visual acuity [24, 25].

The test was chosen as preferred by 87% of children (84/96) and 80% of parents (77/96). The difficulty was less, as shown by the FLACC scale. The eMOVA test is fast, with an average duration of one minute, thus it avoids any risk related to LEDs exposure  [36] . The eMOVA test is inexpensive compared to the price of test projectors or Retinomax, which appears to be the most reliable screening method for all criteria[4]. EMOVA strengths are the measurement of angular visual acuity via Raskin's E, its accessibility and the automation of the test. The mobility of the test and further studies could allow its use by non-medical health professionals in the future and this would be a major step forward for the visual screening of children. The possibility of being self-directed at home by the child or his parents should be explored and could increase screening capacity and at-home monitoring progress in the rehabilitation of amblyopia [28, 34]. The computerization of the data may also have an epidemiological interest [37].

The eMOVA test, however, showed some limitations with a lower concordance for the lowest near visual acuity, but these data are consistent with those in the literature for similar studies [29, 38, 39]. In our sample, only 16% (15/96) of children using eMOVA had their first visit. There was 34% (33/96) strabismus and the prevalence of amblyopia was 19% (18/96) considerng the Rossano-Weiss test as reference in our study. These incidence of amblyopia and strabismus are much higher than those found in the literature [1, 28, 40–57]. This is explained because these children were visiting a specialized ophthalmic-pediatric center. This bias may limit the extrapolation of our results to screening conditions. In addition, most of the children included had already benefited from previous consultations. Only 16% of the children in our sample came for the first time. The remaining 84% were therefore trained in the Rossano-Weiss test, which can create a bias related to a learning effect. However our study has the merit of being a study of real life contrary to what one could find in the literature [24]. The isolated optotype presentation decreases the contour interaction effect and may overestimate visual acuity compared to scales including a linear presentation of the optotypes. The eMOVA test presents the possible choices for pairing in groups of four, but the optotype to be recognized is presented in isolation. This phenomenon could help to explain the overestimation of near visual acuity with the eMOVA test.

Several studies have sought to determine the optimal technical characteristics of tablets for the evaluation of visual acuity and to show the interest of these media [58–60]. Further studies are needed to verify whether the near visual acuity measurements obtained with the eMOVA test can be comparable on tablets of different models and brands and how it could be used at home.

The choice of gold standard can also be discussed. The correct level of concordance found in our study shows the lack of accuracy between the two tests but does not tell us about the test whose measurements are closest to reality. No visual acuity test is perfect and the Rossano-Weiss test has its own flaws. The eMOVA test should be compared to other scales of visual acuity and especially to the ETDRS scale to better define its limits. We have chosen the Rossano Weiss test to provide a measure of angular visual acuity and because we use it in current practice in our center and in most of the ophthalmic-pediatric departments in our country. We could have used the Lea Symbols scale, which presents good performance parameters for children, but it wouldn’t have been an angular assessment of visual acuity or the HOTV test but this requires knowledge of the alphabet which is not possible for 3 years.

Our study led to the development of a simple method for assessing near visual acuity of children on tablets. It revealed a correct match of the eMova test with the reference method we use in common practice, the Rossano-Weiss test. This new test has been successfully submitted to children with a high rate of acceptability from both children and their parents. Further studies are needed to evaluate the value of this test on a larger scale.

## Conclusions

The eMOVA test appears as a reliable method of assessing near visual acuity that could be used both in consultation and in the future on a larger scale in the context of screening as well as for the care of the most difficult children.

## Data Availability

The data directly supporting the publication can be accessed on request to the first author.

## Abbreviations

eMOVA: electronic Measurement Of Visual Acuity
PDA: palmtop computer
AMD: age macular degeneration
FLACC scale: Face Legs Activity Cry Consolability scale
ICC: intra-class correlation coefficient
ETDRS scale: Early Treatment Diabetic Retinopathy Study scale
